# Neuropsychiatric Symptoms in Mild Cognitive Impairment and Dementia Due to AD: Relation With Disease Stage and Cognitive Deficits

**DOI:** 10.3389/fpsyt.2021.707580

**Published:** 2021-08-17

**Authors:** Wietse A. Wiels, Mandy M. J. Wittens, Dieter Zeeuws, Chris Baeken, Sebastiaan Engelborghs

**Affiliations:** ^1^Department of Neurology, Universitair Ziekenhuis Brussel, Brussels, Belgium; ^2^Center for Neurosciences (C4N), Vrije Universiteit Brussel (VUB), Brussels, Belgium; ^3^Department of Biomedical Sciences, University of Antwerp, Antwerp, Belgium; ^4^Department of Psychiatry, Universitair Ziekenhuis Brussel, Brussels, Belgium; ^5^Ghent Experimental Psychiatry (GHEP) Lab, Department of Psychiatry and Medical Psychology, Ghent University Hospital, Ghent University, Ghent, Belgium; ^6^Department of Electrical Engineering, Eindhoven University of Technology, Eindhoven, Netherlands

**Keywords:** behavioral and psychological symptoms of dementia, neuropsychiatric symptoms, mild cognitive impairment, Alzheimer's disease, depressive symptoms, memory clinic

## Abstract

**Background:** The interaction between neuropsychiatric symptoms, mild cognitive impairment (MCI), and dementia is complex and remains to be elucidated. An additive or multiplicative effect of neuropsychiatric symptoms such as apathy or depression on cognitive decline has been suggested. Unraveling these interactions may allow the development of better prevention and treatment strategies. In the absence of available treatments for neurodegeneration, a timely and adequate identification of neuropsychiatric symptom changes in cognitive decline is highly relevant and can help identify treatment targets.

**Methods:** An existing memory clinic-based research database of 476 individuals with MCI and 978 individuals with dementia due to Alzheimer's disease (AD) was reanalyzed. Neuropsychiatric symptoms were assessed in a prospective fashion using a battery of neuropsychiatric assessment scales: Middelheim Frontality Score, Behavioral Pathology in Alzheimer's Disease Rating Scale (Behave-AD), Cohen-Mansfield Agitation Inventory, Cornell Scale for Depression in Dementia (CSDD), and Geriatric Depression Scale (30 items). We subtyped subjects suffering from dementia as mild, moderate, or severe according to their Mini-Mental State Examination (MMSE) score and compared neuropsychiatric scores across these groups. A group of 126 subjects suffering from AD with a significant cerebrovascular component was examined separately as well. We compared the prevalence, nature, and severity of neuropsychiatric symptoms between subgroups of patients with MCI and dementia due to AD in a cross-sectional analysis.

**Results:** Affective and sleep-related symptoms are common in MCI and remain constant in prevalence and severity across dementia groups. Depressive symptoms as assessed by the CSDD further increase in severe dementia. Most other neuropsychiatric symptoms (such as agitation and activity disturbances) progress in parallel with severity of cognitive decline. There are no significant differences in neuropsychiatric symptoms when comparing “pure” AD to AD with a significant vascular component.

**Conclusion:** Neuropsychiatric symptoms such as frontal lobe symptoms, psychosis, agitation, aggression, and activity disturbances increase as dementia progresses. Affective symptoms such as anxiety and depressive symptoms, however, are more frequent in MCI than mild dementia but otherwise remain stable throughout the cognitive spectrum, except for an increase in CSDD score in severe dementia. There is no difference in neuropsychiatric symptoms when comparing mixed dementia (defined here as AD + significant cerebrovascular disease) to pure AD.

## Introduction

Neuropsychiatric symptoms, often called behavioral and psychological symptoms of dementia (BPSD), are highly common in individuals suffering from Alzheimer's disease (AD) (1). They negatively impact quality of life (2) and are often experienced as more burdensome than cognitive manifestations of disease (3). Furthermore, neuropsychiatric symptoms have been identified as not merely symptomatic and themselves carry pathogenic and prognostic weight in cognitive disorders (4–6). Unfortunately, standard therapeutic interventions used in other clinical settings, such as psychotherapy (7), have poor efficacy due to cognitive decline, and pharmacological (8) approaches may even be harmful due to side effects, especially in the elderly (9). Nevertheless, accumulating evidence suggests a role for therapeutic interventions in the treatment and prevention of the underlying neurodegeneration itself, apart from their symptomatic effect (10). This underscores the need for the identification of risk factors for BPSD in order to develop new approaches to prevent and treat these symptoms (11). Moreover, recent evidence has suggested that recognition of neuropsychiatric symptoms may increase detection of cognitive decline in a primary care setting (11). Given the ubiquitous nature of these disorders and the important role of prevention and risk prediction, they deserve specific attention. This underscores the need for the identification of risk factors for BPSD in order to develop new approaches to prevent and treat these symptoms (12).

However, making targeted therapy and prevention more difficult, multiple symptom manifestations are quite common and clustering in groups of symptoms that frequently overlap (13). These include—but are not limited to—delusions, hallucinations, agitation and irritability, aggressiveness, depression, anxiety, apathy, and sleep disturbance. They may occur during the entire disease course, spanning from the prodromal stage to severe dementia (14). As has been examined by several authors, these symptoms are prevalent in AD (15, 16) and dementia in general (17) in multiple settings (e.g., clinical vs. population) (18).

The exact causal mechanisms contributing to these symptoms are varied and complex. For instance, they can be caused by functional, neurochemical, and structural brain changes, occurring in neurodegenerative and cerebrovascular disorders leading to dementia such as AD (19–21), but they may also be impacted by psychological and psychosocial factors as well as premorbid personality traits (22). For a recent discussion of the available high-quality evidence, see Piras et al. (23). Conversely, BPSD have been associated with cognitive decline the other way around (6, 15, 24). Especially in cases of mild cognitive impairment (MCI), a heterogeneous construct that includes AD, non-AD neurodegenerative brain diseases, and other conditions like depression, cause and effect are often hard to disentangle (25). Although some BPSD symptom clusters tend to become more severe over time (26), some others may decrease (27, 28). Other studies have reported more depressive and other behavioral symptoms in cases of dementia with vascular component (20, 29, 30). The following hypotheses were formulated *a priori* in this study: (1) depressive symptoms are more prevalent in MCI and (2) patients with dementia due to AD with significant cerebrovascular disease have a different profile with regard to neuropsychiatric symptoms than patients with pure AD.

Considering all of the above, it is imperative to further investigate the interactions between neuropsychiatric symptoms and cognitive decline in different stages of AD.

## Methods

### Study Cohort

The study population consisted of a total of 779 patients with dementia due to AD and 399 patients with MCI, selected from an existing database as described below. Patients were included at the moment of their diagnostic workup for cognitive decline in a tertiary care level memory clinic between 1996 and 2013 in a prospective fashion (31–33). Study methods are described below.

### Diagnosis

All subjects underwent a general medical and neurological history and physical examination by board-certified neurologists. Standard blood examination and structural neuroimaging (mostly magnetic resonance imaging or computed tomography in case of contraindications for the former) were performed. Probable time since symptom onset was estimated by interviewing the patient's main caregiver and/or legal representative.

Any use of psychotropic drugs was thoroughly investigated by subject and caregiver interview. We defined as psychotropic any use of benzodiazepines and z-drugs, chloral hydrate, antidepressants and antipsychotic drugs of all classes/generations, stimulants, cholinesterase inhibitors, and antiparkinsonian drugs including amantadine. A subject not taking any of these substances in the preceding months was defined as free of psychotropic medication.

The cognitive evaluation was performed by means of a full neuropsychological examination and a Mini-Mental State Examination (MMSE) (34). The general degree of cognitive decline was ascertained using the Global Deterioration Scale (GDetS) (35).

MCI was diagnosed using Petersen's criteria (36): (1) cognitive symptoms, corroborated by an informant; (2) objective cognitive impairment, quantified as a performance of more than 1.5 SD below the appropriate mean on the neuropsychological subtests; (3) largely normal general cognitive functioning; (4) essentially intact activities of daily living (basic and instrumental activities of daily living were determined by an interview with patient and informant); and (5) not demented. Major psychiatric disorders as the cause of cognitive impairment were an exclusion criterion. As all cognitive domains of subjects were tested in an extensive time-linked (±3 months) neuropsychological examination, all MCI patients were categorized as an “amnestic” subtype with memory deficits or a “non-amnestic” subtype with cognitive decline in areas other than memory; cognitive impairment could be present in a “single domain” or in “multiple domains” (37), as described earlier (31).

Probable AD was diagnosed by National Institute of Neurological and Communicative Disorders and Stroke and the Alzheimer's Disease and Related Disorders Association (NINCDS/ADRDA) criteria (38), and subjects also fulfilled the Diagnostic and Statistical Manual of Mental Disorders, Fourth Edition, (DSM-IV) criteria for dementia (39). We defined mixed dementia (MXD) in this cohort as a combination of probable AD and probable or possible vascular dementia (VaD), as diagnosed by the National Institute of Neurological Disorders and Stroke and Association Internationale pour la Recherché et l‘Enseignement en Neurosciences (NINDS/AIREN) criteria for the diagnosis of VaD (40).

All patients and caregivers who consented were followed up clinically, adding to the diagnostic accuracy of our cohort. Multiple subjects underwent, after prior consent, neuropathological examination following autopsy as described previously (41). We thus obtained several “definite” diagnoses in our cohort.

Other specific dementia etiologies [e.g., frontotemporal dementia (FTD), progressive supranuclear palsy, and advanced Parkinson's disease] were diagnosed using the appropriate criteria at the time of diagnosis. These subjects with non-AD dementias were not included in the analyses that this paper reports on.

A cohort of 108 age-matched controls was obtained from an earlier study (32). It consists mainly of spouses of cognitively impaired study participants.

### Neuropsychiatric Evaluation

All patients underwent thorough neuropsychiatric assessment at baseline, including extensive caregiver interviewing. Tests used to evaluate mental and behavioral symptoms were the Middelheim Frontality Score (MFS) (42), Cohen-Mansfield Agitation Inventory (CMAI) (43), Behavioral Pathology in Alzheimer's Disease Rating Scale (Behave-AD) (44), Cornell Scale for Depression in Dementia (CSDD) (45), and 30-item Geriatric Depression Scale (GDS-30) (46).

The MFS is a scale that assesses frontal lobe function and was validated for clinical differentiation between AD and FTD. Information is obtained by interviewing the subject's main caregiver (professional or non-professional) and during an interview of the patient, as well as study of the available clinical files and general behavioral observation. It consists of 10 items to be rated by the clinician or researcher as being either present (1 point) or absent (no point), leading to a total score out of a maximum of 10, as follows: (1) initially comparatively spared memory and spatial abilities; (2) loss of insight and judgment; (3) disinhibition; (4) dietary hyperactivity (referring to overeating); (5) changes in sexual behavior; (6) stereotyped behavior; (7) impaired control of emotions, euphoria, or emotional bluntness; (8) aspontaneity; (9) speech disturbances such as stereotyped phrases, logorrhea, echolalia, mutism, and amimia; and (10) restlessness. A higher score is indicative of more frontal lobe symptoms.

The CMAI is a caregiver's rating questionnaire that assesses 29 different agitated and aggressive behaviors. These are scored on a 7-point scale related to frequency (1 = never to 7 = several times an hour). Subsection scores are available for three clusters of items: aggressive behavior (10 items), physical non-aggressive behavior (11 items), and verbal aggression or agitation (eight items). A higher score means more agitated and/or aggressive behavior.

The Behave-AD is a clinical rating scale for the assessment of pharmacologically remediable neuropsychiatric symptoms in AD. It consists of 25 individual items rated by a 4-point scale of severity from 0 (absent) to 3 (severely troubling to patient or caregiver). Seven groups of symptoms, often called clusters, are assessed: paranoid and delusional ideation (cluster A), hallucinations (cluster B), activity disturbances (cluster C), aggressiveness (cluster D), diurnal rhythm disturbances (cluster E), affective disturbance (cluster F), and anxieties and phobias (cluster G). The “total” score is the sum of all these cluster scores, while the “global” score denotes the impact of behavioral symptoms on caregiver well-being and/or patient safety taken as a whole. A higher score means more troubling neuropsychiatric symptoms.

The CSDD was developed to assess signs and symptoms of major depression in patients with dementia based on an interview with an informant and an interview with the patient. The scale consists of 19 items that are rated as 0 (absent), 1 (present), or 2 (severe). These items focus on five aspects of the depressive syndrome: (A) mood-related signs, (B) behavioral disturbance, (C) physical signs, (D) cyclic functions, and (E) ideational disturbance. A higher score means more depressive symptoms, with a cutoff of 6 generally valid as reflecting a psychiatrist-ascertained diagnosis of depression (47).

The GDS-30 is a self-rating screening instrument for depression in the elderly consisting of 30 yes–no questions on various depressive signs and symptoms. A score of 11 or higher implies mild depression; more than 20, severe depression (46). Despite being created for cognitively healthy older adults, GDS-30 retains its validity in MCI and mild dementia (48).

### Ethics

Data collection started after approval of the study protocol by the local ethics committees of the University of Antwerp and Hospital Network Antwerp (ZNA). All subjects or their legal representatives provided written informed consent for participation in this study.

### Statistical Analysis

The dementia population was stratified by dementia severity in three subgroups by the total MMSE score, with scores equal to or higher than 22 and 12, respectively, implying mild and moderate dementia and scores between 0 and 11 indicating severe dementia based on a paper by Perneczky et al. (49) correlating MMSE score with general dementia severity.

Medication use and gender were compared using chi-square tests both across diagnoses and across severity groups. Other comparisons were obtained using an analysis of variance (ANOVA) with a least significant difference (LSD) *post-hoc* test.

All data were analyzed using SPSS 26 (IBM, Statistical Package for the Social Sciences, Chicago, IL, USA). The significance level was set at *p* < 0.05, two-tailed, for all analyses. In the data presented below, we did not correct for repeated measures, although all significant differences remained statistically significant following Bonferroni correction.

## Results

### Demographics

There are significantly fewer female subjects in the MCI group as compared to the dementia groups. There is a significant (although slight) increase in mean age across groups in parallel with disease severity. There is an expected drop in MMSE score and rise in GDetS across groups. MCI patients use significantly less psychoactive medication than all other groups, as do mild dementia patients as opposed to moderate and severe cases. Results are summarized in Supplementary Table 1.

Subjects with MXD are significantly older than those with dementia due to AD but do not differ otherwise in terms of demographics. Supplementary Table 2 illustrates these findings.

### Behavioral and Psychological Symptoms of Dementia

#### Across Severity Groups

**Figure 1:**
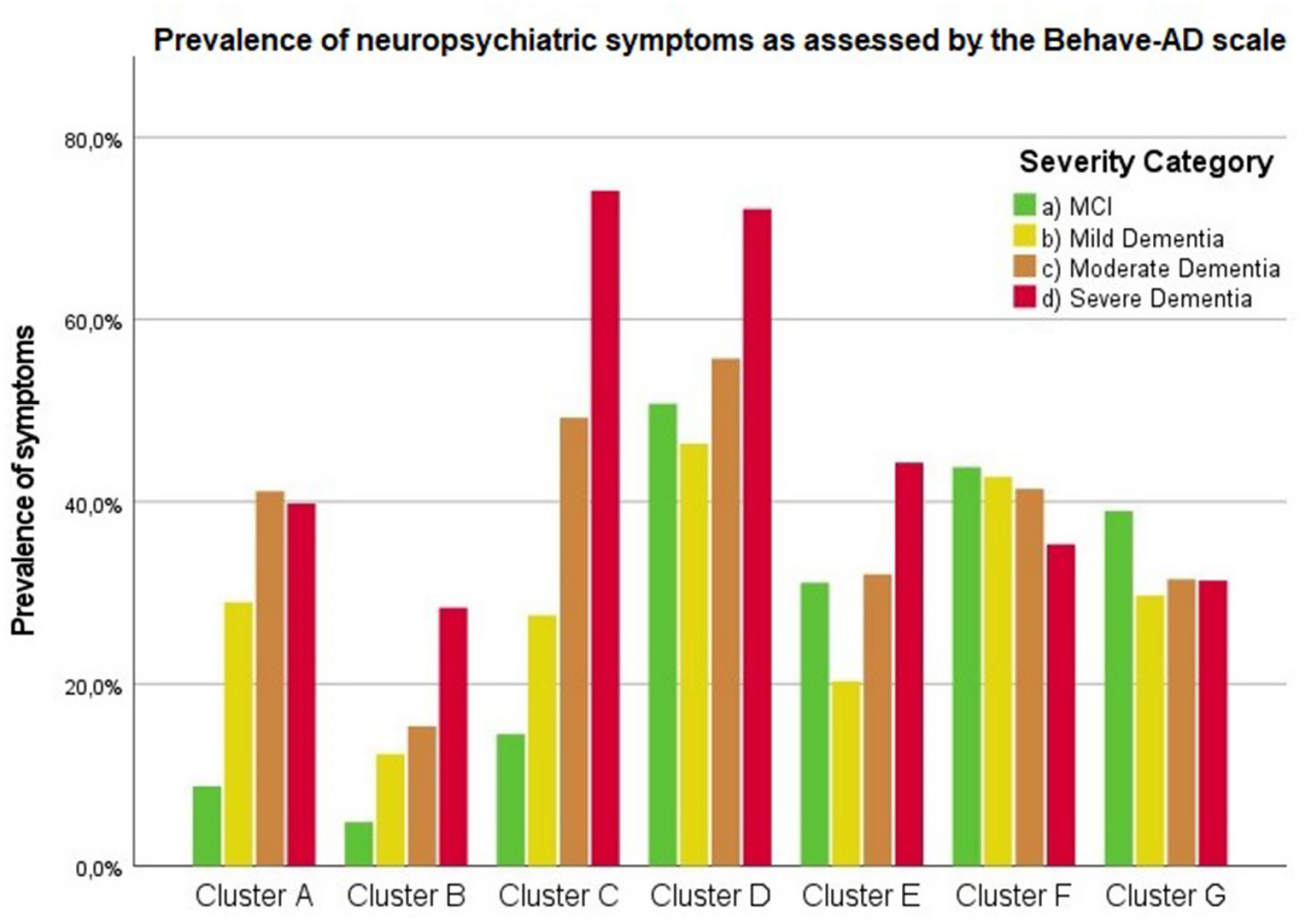
Presence of any neuropsychiatric symptoms as assessed by the Behave-AD scale according to severity of cognitive decline. Cluster A: paranoid and delusional ideation, Cluster B: hallucinations, Cluster C: activity disturbances, Cluster D: aggressiveness, Cluster E: diurnal rhythm disturbances, Cluster F: affective disturbances, Cluster G: anxieties and phobias.

**Figure 2:**
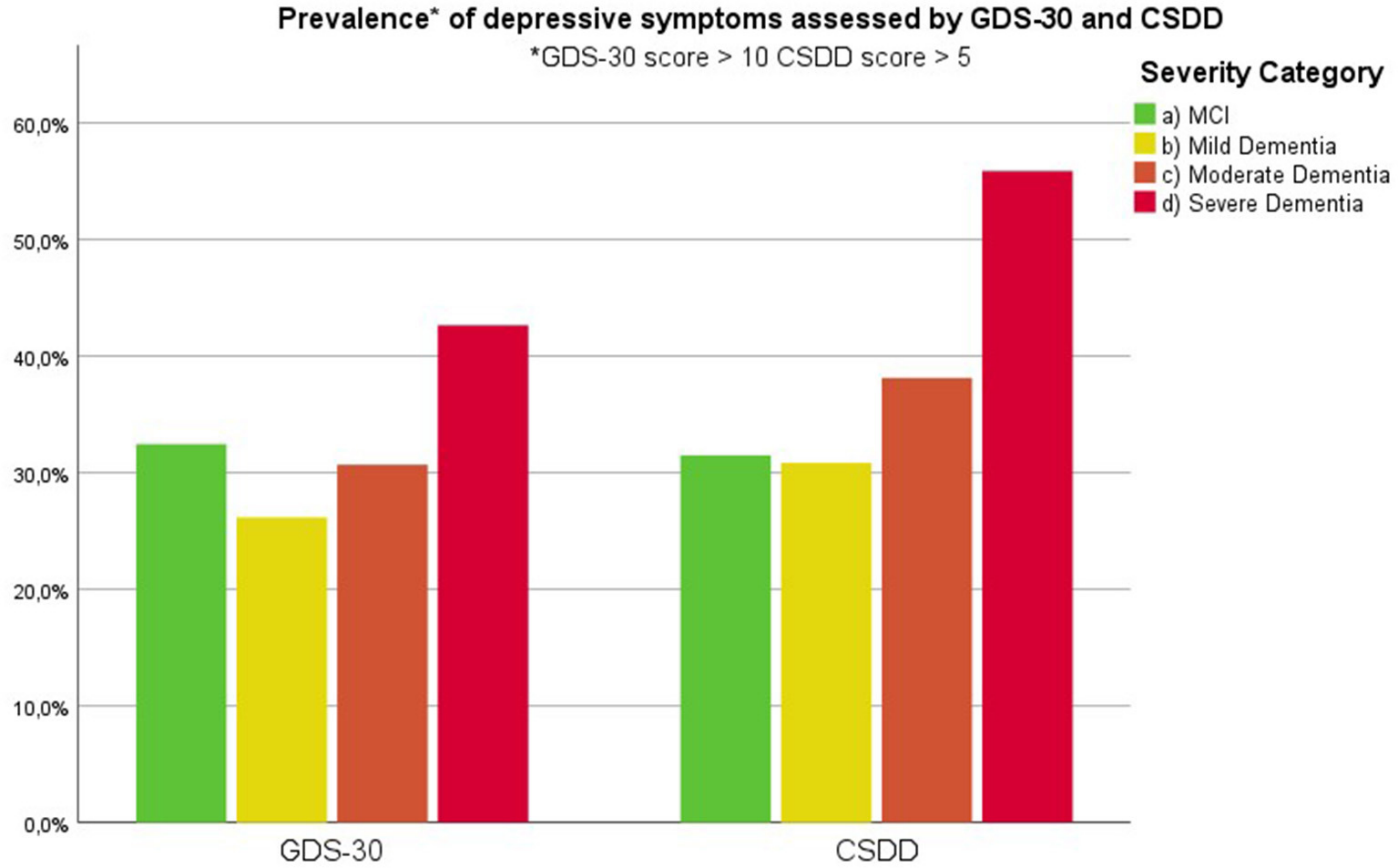
Prevalence of clinically relevant depressive symptoms as assessed by the 30-item Geriatric Depression Scale and Cornell Scale for Depression in Dementia (cut-off value of 10 and 5, respectively) according to severity of cognitive decline.

Findings are summarized in Supplementary Table 3. Figure 1 visualizes the presence of symptoms on the Behave-AD clusters across groups. Figure 2 lists the prevalence of significant depressive symptoms on GDS-30 and CSDD scales. Since no accepted cutoff values for “relevant” symptoms for CMAI and MFS are available in the literature, we did not include results in prevalence visualization.

**Figure 3:**
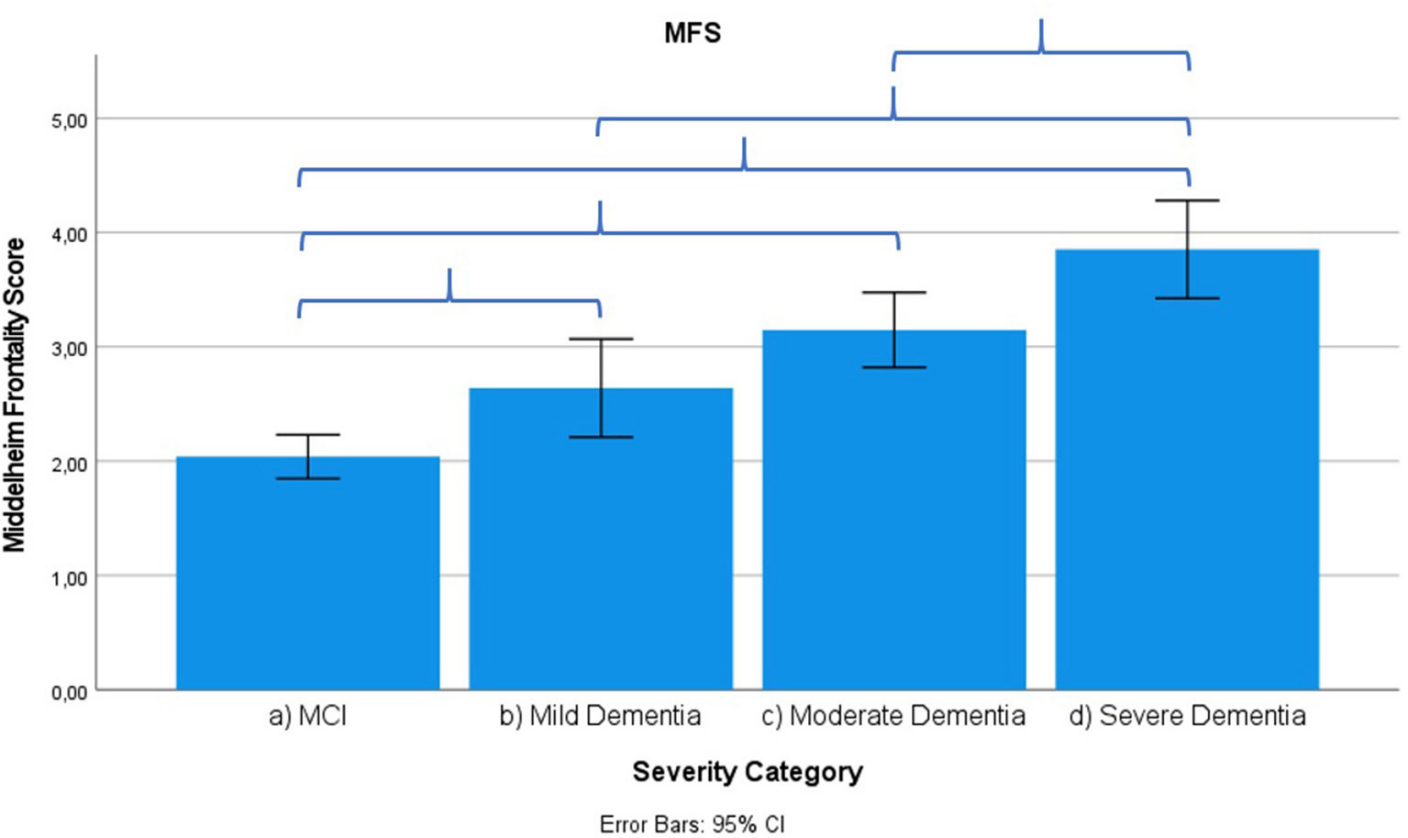
Mean score on the Middelheim Frontality Score (MFS) scale according to severity of cognitive impairment. Error bars are 95% confidence interval. Curly brackets indicate *p* < 0.001.

**Figure 4:**
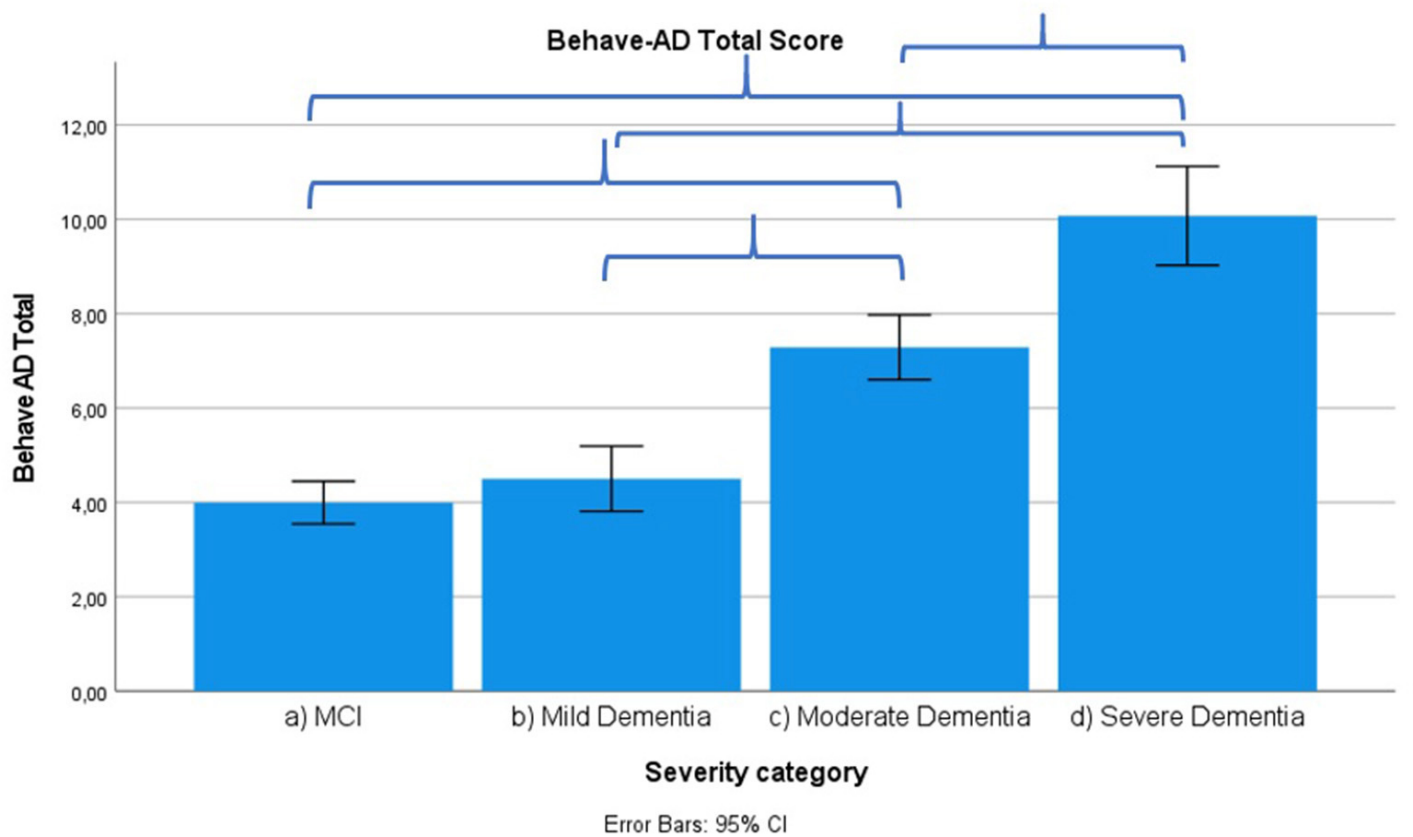
Mean score on the Behave-AD scale total score according to severity of cognitive impairment. Error bars are 95% confidence interval. Curly brackets indicate *p* < 0.001.

**Figure 5:**
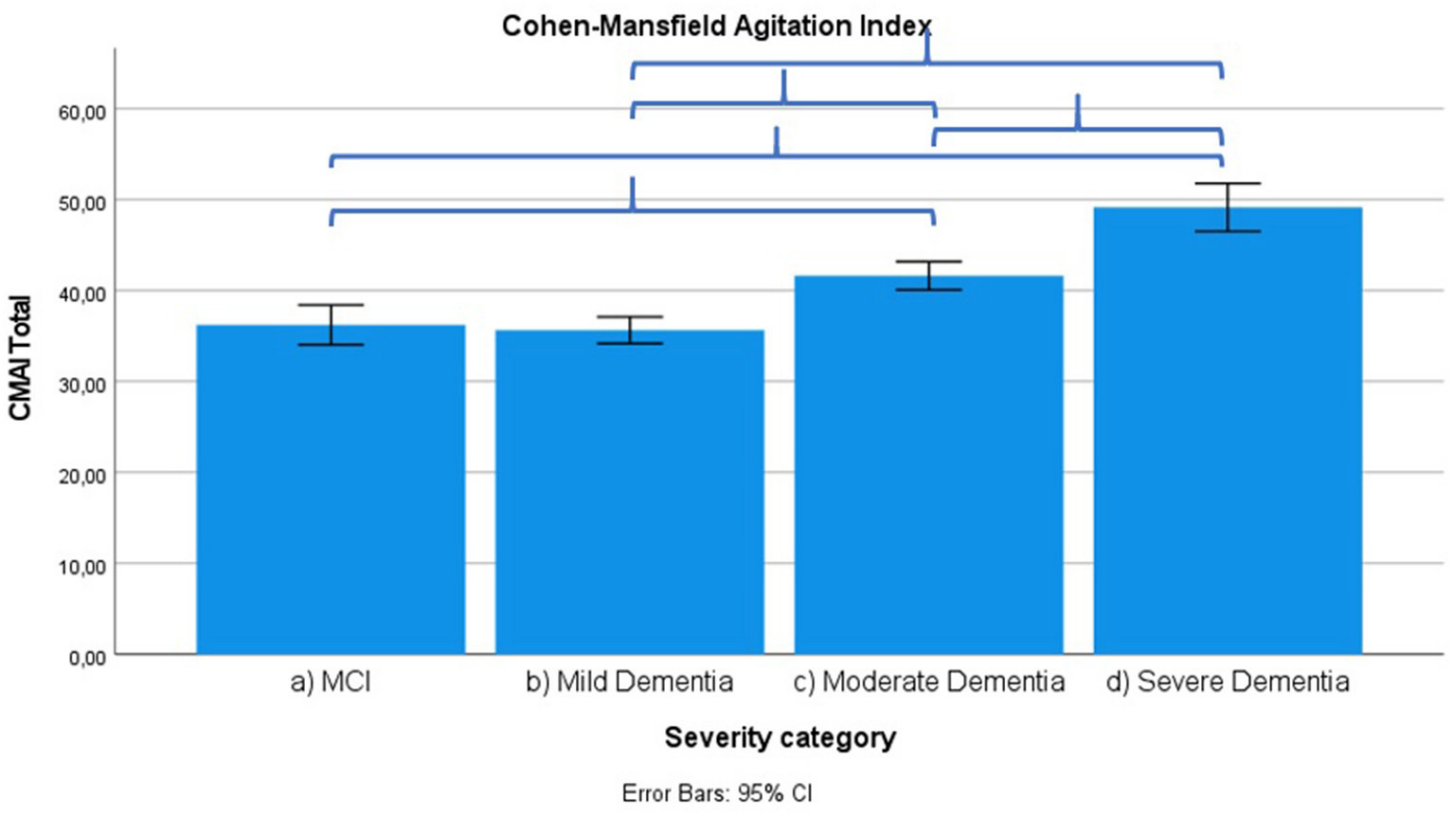
Mean score on Cohen-Mansfield Agitation Index (CMAI) score according to severity of cognitive impairment. Error bars are 95% confidence interval. Curly brackets indicate *p* < 0.001.

**Figure 6:**
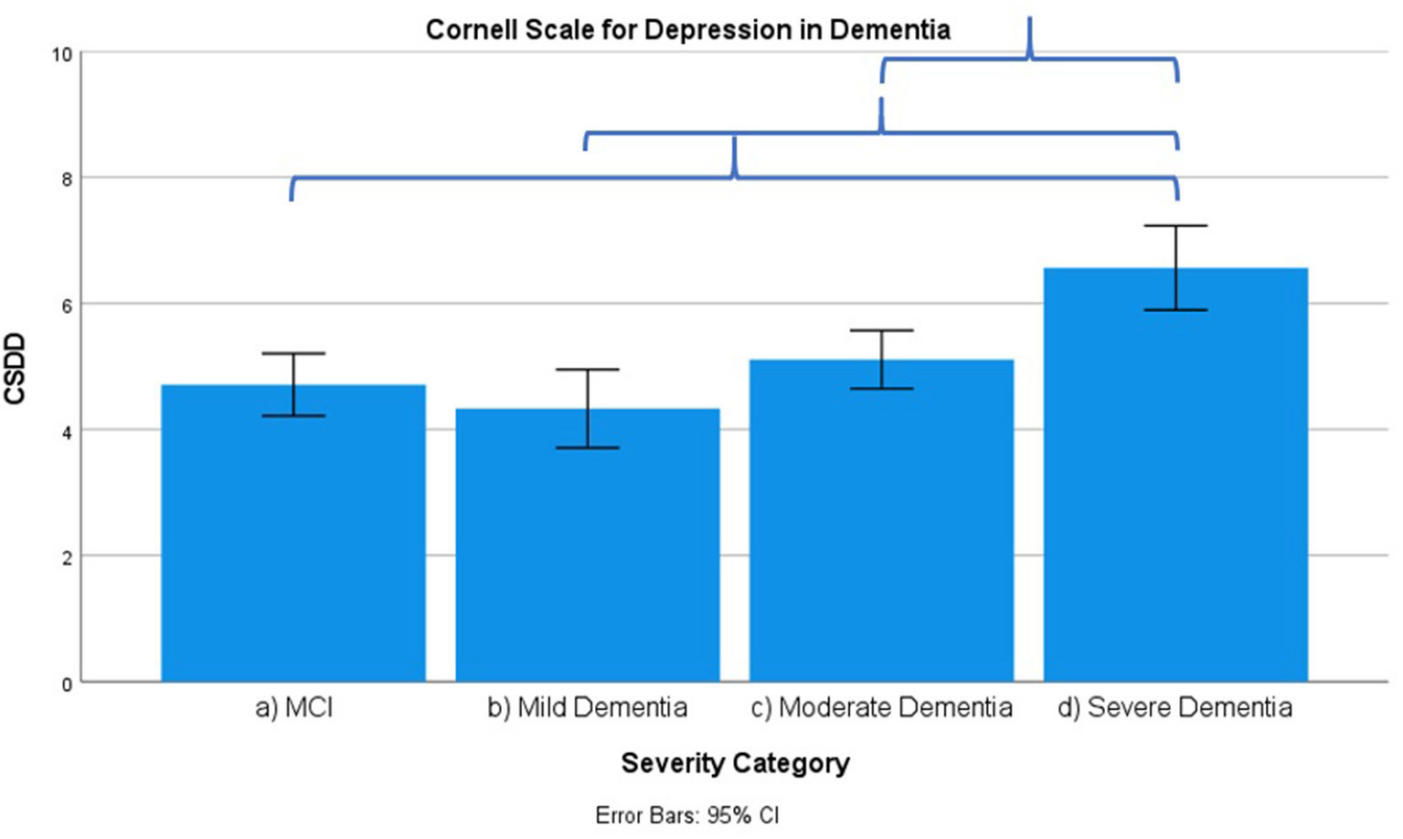
Mean score on the Cornell Scale for Depression in Dementia (CSDD) according to severity of cognitive impairment. Error bars are 95% confidence interval. Curly brackets indicate *p* < 0.001.

**Figure 7:**
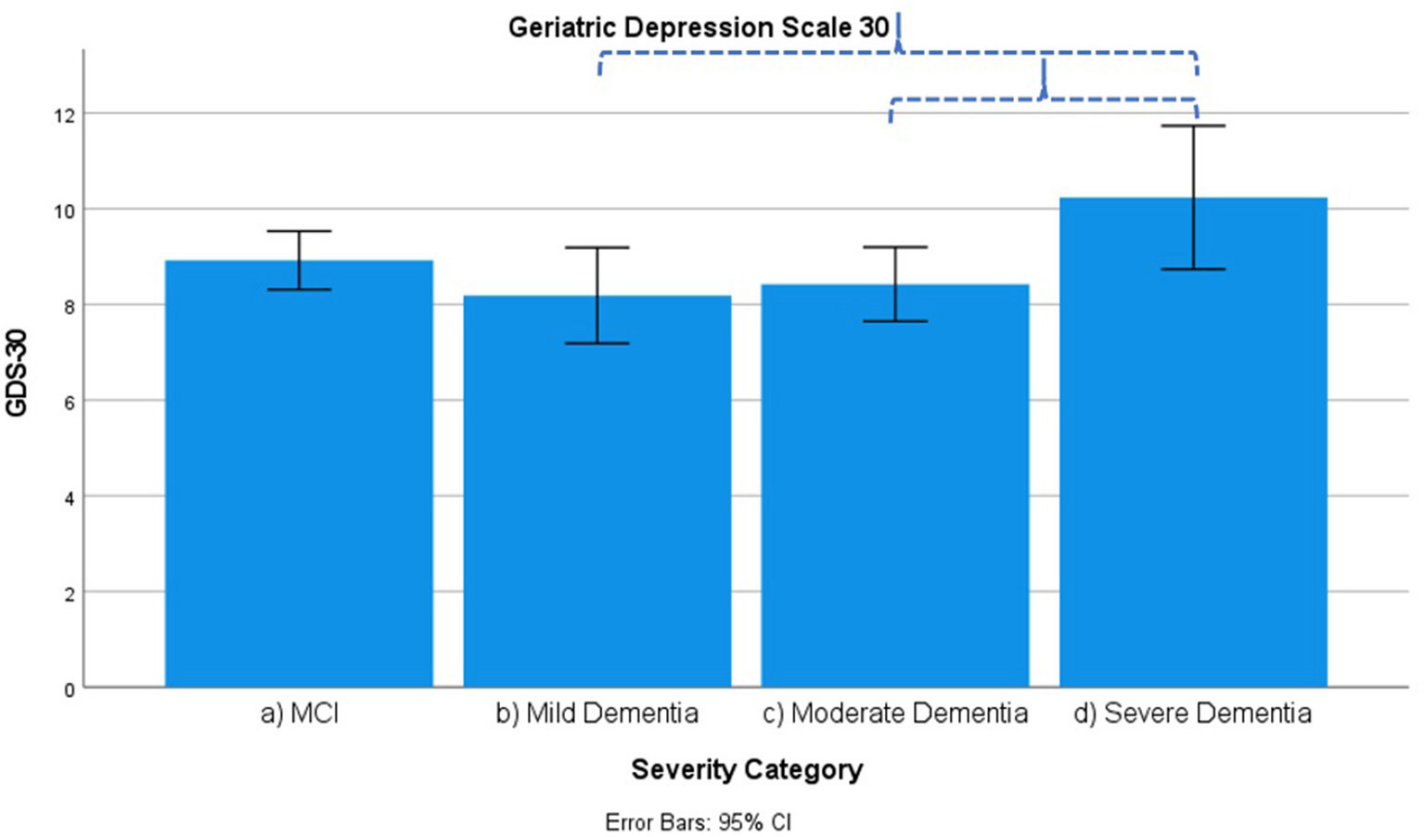
Mean score on the 30 item Geriatric Depression Scale (GDS-30) according to severity of cognitive impairment. Error bars are 95% confidence interval. Dotted curly brackets indicate *p* < 0.05.

Results across severity categories are visually represented in Figures 3–7, with accolades indicating a *p*-value of < 0.001 and dotted accolades representing *p* < 0.05. Separate Behave-AD cluster scores are available in the Supplementary Materials.

Several signs and symptoms become progressively more severe when cross-sectionally (and not longitudinally!) comparing advanced disease stages to earlier ones, as described in what follows.

Frontal lobe symptoms as assessed by the MFS do not differ between mild and moderate dementia. They are more severe in mild dementia compared to MCI, as well as moderate and mild dementia compared to severe dementia. Paranoid and delusional ideations as assessed by Behave-AD cluster A do not differ significantly between moderate and severe dementia but otherwise increase in parallel with increasing severity of cognitive impairment when comparing groups. Hallucinations as assessed by Behave-AD cluster B are significantly worse in the severe dementia stage in comparison with all other groups. They are also more common in moderate dementia when compared to MCI patients. Activity disturbances as assessed by Behave-AD cluster C do not differ in MCI subjects compared to mild dementia. They are more common, however, in moderate dementia and are worse in severe dementia. Aggressiveness as assessed by Behave-AD cluster D does not differ in MCI subjects compared to mild dementia either. They are more common in moderate dementia and very prevalent in severe dementia. The sum of BPSD symptoms as assessed by the Behave-AD total score does not differ significantly between MCI and mild dementia. They do increase in cases of moderate dementia and again in the severe dementia group. The global burden of BPSD as measured by the Behave-AD global score significantly increases when comparing subjects between disease stages. Agitation and aggressiveness as measured by the CMAI and its subclusters do not differ significantly in MCI subjects as compared to mild dementia. They are significantly more common in moderate dementia and even more so in severe dementia. Depressive symptoms as assessed by the CSDD are significantly worse in severe dementia as compared to other groups. Relevant depressive symptoms in CSDD rating (a score of 5 or more) are also more frequent in moderate dementia as compared to MCI and mild dementia.

Some symptoms show a different distribution across severity stages. Note that these are mainly affective symptoms, as described in the following section.

Diurnal rhythm disturbances as assessed by Behave-AD cluster E are more frequent in MCI as compared to mild dementia and similar to moderate dementia. They are slightly worse in severe dementia. Affective symptoms as assessed by Behave-AD cluster F do not differ significantly across groups. Anxiety and phobias as assessed by Behave-AD cluster G are more frequent in MCI as compared to mild dementia but do not differ across the other groups. Depressive symptoms as assessed by GDS-30 trend toward significance when comparing frequency across groups. The presence of relevant symptoms (defined as a score >10) does not differ significantly across groups.

Given the possible effects of gender on the prevalence and severity of neuropsychiatric symptoms (50, 51), we reran all analyses on male-only and female-only cohorts. This did not cause any relevant change in our results. We also evaluated the interaction between gender and neuropsychiatric symptom scores according to severity of cognitive decline. These interactions were not significant (data not shown).

#### Comparing “Pure” Alzheimer's Disease to Mixed Dementia (Vascular Dementia + Alzheimer's Disease)

Results are summarized in Supplementary Table 4. There are slightly more diurnal rhythm disturbances in MXD as compared to AD using the Behave-AD cluster E. MXD patients exhibit more severe depressive symptoms when evaluated using the GDS-30 but not when using the CSDD or Behave-AD cluster F. As the GDS-30 loses validity in severe dementia, the significant difference based on GDS-30 should be interpreted with caution.

## Discussion

### Depression and Anxiety as Related to Severity of Cognitive Decline

Our results indicate that affective symptoms and anxiety are common in MCI, more so than in mild dementia. Depressive symptoms further increase in moderate and severe dementia (Supplementary Table 3; Figures 2, 6, 7).

Depressive symptoms are worse in patients with severe dementia compared to earlier stages of cognitive decline. So does the percentage of subjects with clinically relevant depressive symptoms [defined as a score of 6 or more on the CSDD (47)]. This cutoff is reached by more subjects in the moderate dementia group compared to MCI and mild dementia (Supplementary Table 3–MCI, 32%; mild dementia, 30%; moderate dementia, 38.1%; and severe dementia, 53.6%). These findings suggest that depressive symptoms increase in parallel with the evolution of dementia, discrediting the hypothesis that affective symptoms are common mainly in prodromal and early phases of cognitive decline and decrease later on (27, 52–54).

We should note that this progression of depressive symptoms is less significant when using the GDS-30 scale (Supplementary Table 3; Figure 7). This is probably because of the language-based and metacognitive items included in this self-rated scale, which are difficult to obtain in patients with severe dementia who often lose these cognitive abilities—comprising validity (48). The CSDD was created for use in subjects with (advanced) dementia and therefore has less of a focus on cognitive/affective symptoms compared to physical or behavioral signs and symptoms (47).

In addition to these differences in rating scales, several types of depressive symptoms often coincide throughout the spectrum of cognitive impairment (25, 55, 56). For example, motivational symptoms may overlap with apathy (57, 58) or sedative side effects of psychotropic drugs (9, 59). Vegetative symptoms (60) of depression such as weight loss or other bodily upsets may mimic common somatic issues in the elderly, and *vice versa* (61). Mood disturbances may also be reactional to a diagnosis of cognitive decline [although this is certainly not a universal reaction (62)], being hospitalized and/or moving to a nursing home, etc. All of these may influence the results and interpretation of rating scales assessing several physical (more prominent in the CSDD) and mental (more prominent in the GDS-30) symptoms associated with a depressive syndrome.

There are no differences when using the F cluster of Behave-AD, which consists of an item evaluating tearfulness and an item concerning suicidal thoughts or actions. These may be insensitive as sole markers of depressive symptoms when compared to scales with items assessing more aspects of the depressive syndrome as mentioned above (63, 64).

We found anxiety to be more frequent in MCI patients as compared to advancing stages of dementia. In combination with a relatively high burden of depressive symptoms compared to other neuropsychiatric symptoms (which are rare in MCI), this may reflect the presence of psychiatric disorders as comorbid with MCI (65, 66). Indeed, as major psychiatric disorders as cause of cognitive impairment were an exclusion criterion and as the clinical–diagnostic evaluation was made by a multidisciplinary team consisting of—among others—experienced cognitive neurologists and neuropsychologists, it is unlikely that subjects suffering from major psychiatric disorders (such as clinical depression or anxiety disorders) would have been included. Nevertheless, distinguishing new neuropsychiatric symptoms of degenerative brain disease from preexisting (mild or subsyndromal) psychiatric issues remains challenging (67). Additionally, MCI patients undergoing a diagnostic process of cognitive decline may experience worries about the future (68, 69). As discussed earlier, some authors have suggested that these symptoms may be prodromal to degenerative disease (25, 70, 71) and may wane over time as the underlying brain disease progresses (27). We could not confirm a strongly decreasing trajectory of any neuropsychiatric symptom in this study.

### Other Neuropsychiatric Symptoms as Related to Severity of Cognitive Decline

Our results further demonstrate that there is a gradual progression of most other neuropsychiatric symptoms in parallel with the severity of cognitive decline. This is the case for frontal lobe symptoms, delusions and hallucinations (i.e., psychotic symptoms), activity disturbance, aggressiveness, agitation, and general neuropsychiatric symptoms as measured by the Behave-AD clusters as well as by the Behave-AD total and global scores. Most symptoms are present in a stage of moderate dementia and increase in prevalence and severity in severe dementia. This confirms earlier research suggesting an increasing prevalence and severity of neuropsychiatric symptoms with cognitive decline in AD (41, 42, 72, 73). Of note, some studies have found a decreasing or stable burden in advanced disease (53, 54) which we could not confirm for any of the measures we used. Furthermore, not all neuropsychiatric symptoms linearly increase in frequency or severity throughout the spectrum of cognitive decline. Some neuropsychiatric changes such as depressive symptoms, anxiety, and sleep disruption are present in MCI (Supplementary Table 3). Although we did not statistically compare BPSD data of MCI and dementia patients with healthy controls in this study, our historical control cohort demonstrates that these symptoms are rare in cognitively healthy aging (Supplementary Table 3, column e) (32).

In this large study, psychotropic medication is used a lot more by the dementia groups compared to MCI, even when considering the possible presence of primary or reactive psychiatric disorders among subjects with MCI. In our cohort, only 30% of AD dementia patients were free of psychotropic medication (Supplementary Table 2). One explanation could be that polypharmacy is highly common among nursing home residents with and without dementia (74). Furthermore, subjects undergoing diagnostic evaluation and/or hospitalization related to dementia or delirium are frequently prescribed psychotropic medication (75), despite limited or ambiguous evidence of short- and middle-term efficacy of, for example, antidepressants (76, 77). Nevertheless, some studies have revealed a long-term role for these drugs, implying decreased risk of further cognitive decline under treatment (78). Many studies, however, did not find such an effect (79). As we await stronger evidence, efforts are ongoing to decrease prescribing psychotropic agents that are not stringently indicated (80, 81). Psychotropic drugs may influence behavioral scores. For example, use of benzodiazepines and other sedatives may also mask symptoms of anxiety in subjects in more advanced disease stages. On the other hand, prescription of psychotropics has been linked to increasing care dependence in subjects with dementia (82). A similar explanation may underlie the presence of sleep symptoms as assessed by the Behave-AD section E (consisting of the options: no symptoms, repetitive awakenings, loss of 50–75% of night-time sleep, total loss of night-time sleep/reversal of day–night rhythm), since both depression and anxiety are associated with fragmented sleep and the use of sedative drugs may mask these symptoms. Separately examining subjects with and without use of psychotropic medication in our cohort did not alter our general results concerning differences in mean burden of neuropsychiatric symptoms between groups according to severity of cognitive decline (data not shown). This may reflect an effect of pharmacological treatment on these symptoms or the heterogeneity of these subjects, since several kinds of psychotropic drugs were considered together.

As mentioned above, the effect of gender on all of the above was minimal in our cohort after separate analysis of male and female subjects as well as an analysis of interaction between gender, neuropsychiatric symptom scores, and severity of cognitive decline. This did not yield any significant results (not shown here).

Summarizing, our results indicate that most behavioral symptoms such as psychosis, aggression, and activity disturbance increase linearly with advancing dementia. We further demonstrate that affective symptoms are frequent in MCI and seem to remain stable throughout the course of cognitive decline in AD. Depressive symptoms are common and increase with the severity of dementia.

### Neuropsychiatric Symptoms as Related to Dementia Diagnoses (Alzheimer's Disease vs. Mixed Dementia)

We did not observe significant differences between the AD and MXD groups in our cohort, barring a slightly higher incidence of (relevant) depressive symptoms as assessed by the GDS-30, which was not reproduced using other measures of depressive symptoms. This may be due to our definition of MXD, which is probable AD in combination with significant cerebrovascular disease. Differences with cohorts comprising “pure” VaD may be higher, as has been suggested in research on late-life depressive symptoms and vascular disease (19, 20, 30). Although not all studies have confirmed this link (83), systematic literature review does suggest such a relation (84). Causal mechanisms, however, remain controversial. They may include structural damage to neural networks (30, 85) or a shared inflammatory pathogenesis (86). Much research in this focuses on “pure” vascular/subcortical dementia, focusing, for example, on white matter lesions (87–89). Much fewer studies have explicitly evaluated neuropsychiatric profiles of MXD vs. AD (90, 91).

As mentioned before, certain depression scales such as the GDS-30 lose validity in severe dementia (48), which may bias our findings. Nevertheless, an absence of significant differences between AD and MXD subjects remained even when removing cases of severe dementia from the analysis (not shown here). Our study's results therefore suggest that considering “pure” AD together with instances of AD with significant cerebrovascular disease is justifiable when studying neuropsychiatric symptoms.

### Limitations

Like all diagnostic instruments, the rating scales used in our study have intrinsic limitations. It has, for example, been suggested that the CMAI is prone to proxy reporting bias due to the highly distressing nature of these symptoms for caregivers (92). We discussed the possible limitations of the GDS-30 in severe dementia earlier.

An important limitation of this study is the absence of apathy measures. Despite phenomenological overlap with depression concerning, for example, loss of interest or motivation (93), apathy is a distinct neuropsychiatric syndrome (94). It has been increasingly recognized as an important predictor of dementia in both community-dwelling (95) and clinical settings—with and without concurrent depressive symptoms (96–98). Apathy has furthermore been associated with important reduction of quality of life in patients as well as caregivers (99, 100).

Additionally, our study evaluated depressive symptoms in a cross-sectional fashion. Although a thorough medical history including psychiatric disorders (including depression) was obtained from all participants, we did not systematically assess the frequency and severity of past depressive episodes or duration of current affective symptoms. It has been suggested that new-onset or worsening depressive symptoms in the elderly are related to cognitive decline (4). A lifetime history of depression has also been implicated in dementia risk (25). This lack of temporal information on affective symptoms is a limitation, since it is known to be of diagnostic and prognostic importance. It has been argued that new and increasing affective symptoms especially increase the risk of dementia (25, 101, 102). This a limitation of this study and must caution possible conclusions.

Notwithstanding the prospective nature of our neuropsychiatric assessment, it has been reported that memory clinic cohorts have more severe neuropsychiatric symptoms (103, 104) as opposed to individuals not seeking medical attention. The presence of these symptoms may have caused subjects or their caregivers to seek professional help earlier, which could have caused an overestimation of the prevalence of these symptoms, resulting in a selection bias. Our results should therefore be extrapolated to non-clinical populations with caution.

We did not analyze marital status, estimated duration of cognitive symptoms, and level of education. A more thorough subtyping of the impact of specific classes of psychotropic drugs on our findings was not done. We considered the use of several psychotropic drugs together in a binary fashion; this may explain the lack of impact on our findings as mentioned above. Lastly, we did not evaluate the role of imaging, genetic, or biochemical [i.e., cerebrospinal fluid (CSF) biomarker] data in this study.

## Conclusion

Depressive symptoms are prevalent in MCI. They increase in severity and prevalence in moderate dementia and in severe dementia. Anxiety is frequent in MCI and remains roughly stable throughout the cognitive spectrum. Frontal lobe symptoms, psychosis, agitation, and activity disturbance worsen linearly as cognition declines. There is no clear difference in neuropsychiatric symptoms when comparing pure AD to mixed vascular-AD dementia.

Neuropsychiatric symptoms are highly common in moderate and severe dementia despite frequent pharmacotherapy, demonstrating a clear need for new therapeutic options for these incapacitating symptoms.

## Data Availability Statement

The raw data supporting the conclusions of this article will be made available by the authors, without undue reservation.

## Ethics Statement

The studies involving human participants were reviewed and approved by Ethical Committees of University of Antwerp and Hospital Network Antwerp (ZNA). The patients/participants provided their written informed consent to participate in this study.

## Author Contributions

WW, CB, and SE conceived the idea for this manuscript. WW performed the database analysis with help from MW and DZ. WW wrote the first drafts. MW, DZ, CB, and SE critically reviewed and commented on these drafts. All authors read and approved the submitted version.

## Conflict of Interest

The authors declare that the research was conducted in the absence of any commercial or financial relationships that could be construed as a potential conflict of interest.

## Publisher's Note

All claims expressed in this article are solely those of the authors and do not necessarily represent those of their affiliated organizations, or those of the publisher, the editors and the reviewers. Any product that may be evaluated in this article, or claim that may be made by its manufacturer, is not guaranteed or endorsed by the publisher.
