# Supplementary material for: Neuropsychiatric Symptoms in Mild Cognitive Impairment and Dementia Due to AD: Relation With Disease Stage and Cognitive Deficits
Source: Front Psychiatry. 2021 Aug 17;12:707580. doi: 10.3389/fpsyt.2021.707580 (PMC8415837; doi:10.3389/fpsyt.2021.707580)
Supplement: Supplementary file 1 [file Table_1.DOCX]

Tables: Neuropsychiatric symptoms in mild cognitive impairment and dementia due to AD: relation with disease stage and cognitive deficits

|  | a) MCI | b) MILD DEMENTIA | c) MODERATE DEMENTIA | d) SEVERE DEMENTIA | STATISTICS |
| --- | --- | --- | --- | --- | --- |
| %F | 54,0% bc | 64,5% a | 66,4% a | 58,7% | **p =0,003** |
| Age at Baseline | 75,2 (9,0) BCD | 78,1 (7,3) AC | 81,0 (6,6) ABd | 79,4 (7,8) Ac | **p <0,001** |
| MMSE | 26,1 (2,8) BCD | 24,4 (2,0) ACD | 16,6 (2,8) ABD | 5,7 (3,9) ABC | **p <0,001** |
| GDetS | 3,1 (0,8) BCD | 4,0 (0,9) ACD | 5,0 (0,9) ABD | 6,0 (0,6) ABC | **p <0,001** |
| %Free of Psychotropic Medication | 80,7% BCD | 44,2% ACD | 30,5% AB | 33,3% AB | **p <0,001** |

*Table 1 – Severity categories: demographics of severity groups (In % or mean and standard deviation, between brackets). Chi square test for comparisons of gender/medication, one-way ANOVA w/ LSD post-hoc for others. Subscript letters denote categories from which value differs at the 0,05 level for posthoc test. Caps for significance <0,001. a: MCI, b: Mild AD dementia, c: Moderate AD dementia, d: Severe AD dementia. MMSE: Mini Mental State Examination. GDetS: Global Deterioration Scale.*

|  | a) AD DEMENTIA | b) MIXED DEMENTIA | STATISTICS |
| --- | --- | --- | --- |
| %F | 64% | 60% | p=0,535 |
| Age at Baseline | 79,6 (7,2) | 81,6 (6,6) | **p=0,003** |
| MMSE | 15,3 (7,1) | 14,3 (7,2) | p=0,141 |
| GDetS | 5,0 (1,1) | 5,1 (1,0) | p=0,470 |
| % Free of Psychotropic Medication | 35,0% | 30,0% | p=0,206 |

| *Table 2 – Dementia diagnoses: demographics of dementia types (In % or mean and standard deviation, between brackets). Chi square test for comparisons of gender/medication, one-way ANOVA hoc for others. MMSE: Mini Mental State Examination. GDetS: Global Deterioration Scale.* |
| --- |

|  | a) MCI | b) MILD DEMENTIA | c) MODERATE DEMENTIA | d) SEVERE DEMENTIA | e) CONTROL | STATISTICS |
| --- | --- | --- | --- | --- | --- | --- |
| MFS | 2,00 (1.58) BCD | 2,82 (1,73) AD | 3,31 (1.76) AD | 4,10 (1,76) ABC | 0,9 (1,1) | **p <0,001** |
| Behave AD A | 0,18 (0,80) BCD | 0,75 (1,53)ACD | 1,39 (2,27) AB | 1,70 (3,04) AB | 0 (0) | **p <0,001** |
| Behave AD B | 0,12 (0,64) CD | 0,27 (0,85) D | 0,39 (1,15) AD | 0,63 (1,22) ABC | 0,1 (0,4) | **p <0,001** |
| Behave AD C | 0,24 (0,69) CD | 0,58 (1,23) CD | 1,41 (1,85) ABD | 2,53 (2,26) ABC | 0 (0,2) | **p <0,001** |
| Behave AD D | 1,42 (1,87) CD | 1,26 (1,81) CD | 1,99 (2,40) ABD | 3,21 (3,06) ABC | 0,6 (1,1) | **p <0,001** |
| Behave AD E | 0,44 (0,74) bd | 0,28 (0,63) acD | 0,49 (0,83) bd | 0,63 (0,84) aBc | 0,4 (0,6) | **p <0,001** |
| Behave AD F | 0,83 (1,16) | 0,86 (1,20) | 0,91 (1,32) | 0,76 (1,23) | 0,6 (0,9) | p =0,58 |
| Behave AD G | 0,74 (1,22) b | 0,49 (0,96) a | 0,69 (1,33) | 0,62 (1,19) | 0.5 (0.8) | p =0,22 |
| Behave AD Total | 3,99 (4,18) CD | 4,50 (4,10) CD | 7,29 (6,85) ABD | 10,07 (7,55) ABC | 2,2 (2,1) | **p <0,001** |
| Behave AD Global | 0,49 (0,77) BCD | 0,85(0,79)ACD | 1,09 (0,88) ABD | 1,50 (0,94) ABC | 0,0 (0,1) | **p <0,001** |
| CMAI 1 | 10,12 (1,00) CD | 10,23 (1,23) D | 11,27 (4,14) AD | 14,38(8,58) ABC | 10 (0) | **p <0,001** |
| CMAI 2 | 12,27 (2,99) bCD | 13,72 (4,94) aCD | 16,80 (7,32)ABD | 20,56(8,64) ABC | 11,3 (1,0) | **p <0,001** |
| CMAI 3 | 11,64 (4,99) CD | 11,72 (4,85) cD | 13,56 (7,16) Ab | 14,24 (7,46) AB | 11,3 (1,0) | **p <0,001** |
| CMAI Total | 36,23 (10,64) CD | 35,65(8,60) CD | 41,63 (15,31) ABD | 49,15 (18,86) ABC | 30,3 (2,3) | **p <0,001** |
| Cornell SDD | 4,71 (4,55) D | 4,33 (3,57) D | 5,11 (4,28) D | 6,56 (4,15) ABC | 2,9 (2,9) | **p <0,001** |
| DEPR+ (CSDD) | 32% CD | 30% CD | 38,1% ABD | 53,6% ABC | / | **p <0,001** |
| GDS-30 | 8,92 (5,46) | 8,19 (5,25) d | 8,42 (5,77) d | 10,24 (6,19) bc | / | p =0,077 |
| DEPR+  (GDS-30) | 32,6% | 26,0% | 30,6% | 42,6% | / | p=0,164 |

*Table 3: behavioural scores of severity groups. Chi square test for comparison of gender/medication, one-way ANOVA w/ LSD post-hoc for others. Subscript letters denote categories from which value differs at the 0,05 level for posthoc test. Caps for significance <0,001. a: MCI, b: Mild AD dementia, c: Moderate AD dementia, d: Severe AD dementia, e: historical control cohort*^22^ *MFS: Middelheim Frontality Score. CMAI: Cohen-Mansfield Agitation Index. CSDD: Cornell Scale for Depression in Dementia. GDS-30: 30-item Geriatric Depression scale. DEPR+: above the cut-off for clinically relevant symptoms, a score of 5 for the CSDD and 10 for the GDS-30*

|  | a) AD DEMENTIA | b) MIXED DEMENTIA | c) CONTROL | STATISTICS |
| --- | --- | --- | --- | --- |
| MFS | 3,43 (1,84) | 3,09 (1,84) | 0,9 (1,1) | p=0,066 |
| Behave AD A | 1,38 (2,47) | 0,93 (1,80) | 0 (0) | p=0,053 |
| Behave AD B | 0,42 (1,13) | 0,50 (1,12) | 0,1 (0) | p=0,518 |
| Behave AD C | 1,51 (2,01) | 1,55 (1,86) | 0 (0) | p=0,857 |
| Behave AD D | 2,26 (2,6) | 1,92 (2,55) | 0,6 (1,1) | p=0,183 |
| Behave AD E | 0,45 (0,76) | 0,62 (0,88) | 0,4 (0,6) | **p=0,022** |
| Behave AD F | 0,89 (1,28) | 0,74 (1,26) | 0,6 (0,9) | p=0,253 |
| Behave AD G | 0,67 (1,25) | 0,51 (1,07) | 0.5 (0.8) | p=0,190 |
| Behave AD Total | 7,59 (6,96) | 6,79 (6,01) | 2,2 (2,1) | p=0,232 |
| Behave AD Global | 1,19 (0,91) | 1,09 (0,89) | 0,0 (0,1) | p=0,256 |
| CMAI 1 | 11,92 (5,97) | 11,92 (4,69) | 10 (0) | p=0,999 |
| CMAI 2 | 17,37 (7,97) | 16,63 (7,01) | 11,3 (1,0) | p=0,336 |
| CMAI 3 | 13,44 (6,86) | 13,43 (7,04) | 11,3 (1,0) | p=0,983 |
| CMAI total | 42,73 (16,38) | 41,98 (15,58) | 30,3 (2,3) | p=0,637 |
| Cornell SDD | 5,19 (4,02) | 5,82 (4,69) | 2,9 (2,9) | p=0,158 |
| DEPR + (CSDD) ? | 39% | 44% | / | p=0,360 |
| GDS-30 | 8,31 (5,49) | 10,69 (7,11) | / | **p=0,005** |
| DEPR + (GDS-30) ? | 29% | 43% | / | **p=0,043** |

*Table 4: behavioural scores of dementia diagnoses. Chi square for gender/medication, one-way ANOVA for others. Control group from*^22^ *MFS: Middelheim Frontality Score. CMAI: Cohen-Mansfield Agitation Index. CSDD: Cornell Scale for Depression in Dementia. GDS-30: 30-item Geriatric Depression scale. DEPR+: above the cut-off for clinically relevant symptoms, a score of 5 for the CSDD and 10 for the GDS-30*
